# Analysis of the Sequence Characteristics of Antifreeze Protein

**DOI:** 10.3390/life11060520

**Published:** 2021-06-03

**Authors:** Yu-Hang Zhang, Zhandong Li, Lin Lu, Tao Zeng, Lei Chen, Hao Li, Tao Huang, Yu-Dong Cai

**Affiliations:** 1 School of Life Sciences, Shanghai University, Shanghai 200444, China; reyhz@channing.harvard.edu; 2 Channing Division of Network Medicine, Brigham and Women’s Hospital, Harvard Medical School, Boston, MA 02115, USA; 3 College of Food Engineering, Jilin Engineering Normal University, Changchun 130052, China; lizd591@jlenu.edu.cn (Z.L.); lihao@jlenu.edu.cn (H.L.); 4 Department of Radiology, Columbia University Medical Center, New York, NY 10032, USA; ll2860@cumc.columbia.edu; 5 Bio-Med Big Data Center, CAS Key Laboratory of Computational Biology, Shanghai Institute of Nutrition and Health, Chinese Academy of Sciences, Shanghai 200031, China; zengtao@sibs.ac.cn; 6 College of Information Engineering, Shanghai Maritime University, Shanghai 201306, China; lchen@shmtu.edu.cn; 7 CAS Key Laboratory of Tissue Microenvironment and Tumor, Shanghai Institute of Nutrition and Health, Chinese Academy of Sciences, Shanghai 200031, China

**Keywords:** antifreeze protein, protein domain, minimum redundancy maximum relevance, random forest

## Abstract

Antifreeze protein (AFP) is a proteinaceous compound with improved antifreeze ability and binding ability to ice to prevent its growth. As a surface-active material, a small number of AFPs have a tremendous influence on the growth of ice. Therefore, identifying novel AFPs is important to understand protein–ice interactions and create novel ice-binding domains. To date, predicting AFPs is difficult due to their low sequence similarity for the ice-binding domain and the lack of common features among different AFPs. Here, a computational engine was developed to predict the features of AFPs and reveal the most important 39 features for AFP identification, such as antifreeze-like/N-acetylneuraminic acid synthase C-terminal, insect AFP motif, C-type lectin-like, and EGF-like domain. With this newly presented computational method, a group of previously confirmed functional AFP motifs was screened out. This study has identified some potential new AFP motifs and contributes to understanding biological antifreeze mechanisms.

## 1. Introduction

Antifreeze protein (AFP) is a proteinaceous compound with improved antifreeze ability in organisms and is widely identified and validated in various species and subtypes [1,2]. As indicated by its name, this compound inhibits the damage of frozen water/ice on living organisms, and even a small amount of this surface-active material already has a tremendous influence on the growth of ice. How AFPs combine with ice was attempt explained from the following viewpoints: (1) “dipole-dipole” hypothesis model [3], (2) hydrogen atom binding model [4], and (3) rigid body energy theory [5]. Given that AFPs have unique biochemical properties as frost resisting regulators, multiple biochemical, biotechnological, and agricultural production processes have relied on the application of such protein clusters.

In agriculture, AFPs are applied to maintain crop and fish production in colder climates and as a clinical treatment on hypothermia and cryosurgery against tumors, thereby implying its specific role in biotechnological fields [6,7].

AFPs are a group of effective proteins with unique antifreeze properties and typical structural and functional characteristics. From the molecular structural aspect, the current AFPs have originated from the sugar-bind domain of C-type lectins (CTLs) that usually depend on calcium ions. Despite the detailed classification of AFPs, their molecular structures still vary; some common structures are shared by almost all AFPs, and some are also found in the original CTLs such as disulfide bridges and cysteine residues [8]. This finding indicates that different AFPs may have similar evolutionary procedures, and their structures may be essential for their antifreeze property. This concept provides structural foundations for novel AFP predictions.

Considering that AFPs share similar structural properties and are important in the maintenance of biological activity under certain extreme environments, understanding how these materials utilize their typical structural properties to protect living organisms from low temperature and identifying novel AFPs are the two most crucial research directions in this field. Previous studies have partially revealed the biochemical mechanisms underlying AFP protection of living organisms from internal ice formation. AFPs help regulate the freezing point of certain aqueous solutions to provide a soluble and liquid microenvironment for essential biochemical reactions as firstly reported and confirmed in Dendroides canadensis [9]. In 2014, a detailed mechanism study used the Ginzburg–Landau-type approach to identify and validate a specific phenotype as the phase separation of a typical dynamic two-component system and confirmed that AFPs can inhibit the general water freezing procedures in vitro [10].

Depending on their specific molecular structures, AFPs have varying participation in regulating the antifreeze properties of organisms. One of the largest obstacles in current AFP studies is the prediction of novel potential AFPs. Although AFPs share some simple structures such as disulfide bridges and cysteine residues, their sequence similarity is low. Hence, predicting novel AFPs according to previous computational methods is difficult. In this study, a computational engine is developed to predict AFP features. The 39 most important features for AFP identification have been revealed. Candidate features and signatures are extracted from a website called “InterPro protein sequence analysis & classification”. These entries are converted to their protein domain by relying on features that represent protein families, domains, repeats, and specific functional sites. Based on the 39 features, the most common protein domains are antifreeze-like/N-acetylneuraminic acid synthase C-terminal, insect AFP motif, and CTL-like domain. This finding indicates that many important key features originated from the protein domains such as insect AFP motif and CTL-like domain. In addition, a group of previously confirmed functional AFP motifs is screened out by using the newly presented computational method. This study has identified some potential new AFP motifs and contributes to the understanding of biological antifreeze mechanisms.

## 2. Materials and Methods

### 2.1. AFPs and Their Sequence Features

A total of 9083 non-AFP and 464 AFP sequences were downloaded from Yang et al. [11] and are provided in Supplementary Material S1. InterProScan was used to scan and annotate the sequence characteristics of these proteins [12]. Proteins without domain annotations were removed. Finally, a matrix with proteins as rows (420 “1” meant AFP and 8998 “2” meant non-AFP) and 13,729 domains as columns was established. If a protein, i, has a domain, j, then the value in a row, i, and column, j, is 1; otherwise, it is 0. The protein domain matrix is provided in Supplementary Material S2. The AFPs were termed as positive samples, whereas non-AFPs were called negative samples.

### 2.2. Minimum Redundancy Maximum Relevance Feature Selection

Lots of domains were used to encode one AFP or non-AFP. Not all domains were highly related to the determination of AFPs. To extract highly related domains, thereby building an efficient classifier, a feature selection procedure was necessary. Here, we selected the widely used minimum redundancy maximum relevance (mRMR) [13,14,15,16] feature selection method. Such a method evaluates the importance of features using maximum relevance and minimum redundancy. The former indicates that features that are highly related to class labels are important, whereas the latter one means features that have low redundancies to other features are also important. The relevance and redundancy are all used mutual information (MI) to quantify. For two variables *x* and *y*, their MI value can be computed by

(1) $$I(x,y)=\iint p(x,y)\log\frac{p(x,y)}{p(x)p(y)}dxdy,$$

where *p*(*x*, *y*) is their joint probabilistic density, and *p*(*x*) and *p*(*y*) are their marginal probabilistic densities. The mRMR method outputs a feature list, named mRMR feature list, to indicate the importance of features. At first, this list is empty. Features are selected one by one and appended to this list. For each of the non-selected features, calculate its relevance to class labels, measured by MI of it and class labels, and its redundancies to already-selected features, evaluated by the average MI values of it and each already-selected feature. The difference between the relevance and redundancies is further computed and a feature with a maximum difference is picked up. This procedure stops until all features have been selected. For formulation, the feature list is denoted as

(2) $$F=[f_{1},f_{2},\ldots ,f_{N}],$$

where *N* is the number of all input features. Features that have high ranks are more important than those with low ranks.

### 2.3. Incremental Feature Selection

The mRMR method produced an mRMR feature list. According to the idea of mRMR, some top features can comprise the optimum feature subsets for a given classification algorithm. Thus, the IFS method [17] was employed to extract discriminative features with the best performance for one classification algorithm (e.g., random forest (RF) [18]). In accordance with the mRMR feature list *F*, a series of feature subsets $F_{1},F_{2},\ldots F_{N}\;$was generated, where $F_{1}=\left\{f_{1}\right\}$, $F_{2}=\left\{f_{1},f_{2}\right\}$, and so forth. A classifier can be built on each feature subset, where samples were represented by features in such a list. Then, 10-fold cross-validation [19,20,21,22,23,24,25,26] was adopted to evaluate the performance of all classifiers. Finally, the classifier with the best performance can be found and the corresponding feature subset was extracted. Such classifier was termed as the optimum classifier and features in the corresponding feature subset were called optimum features.

### 2.4. Random Forest

As mentioned in Section 2.3, the IFS method needed a classification algorithm. Here, we selected the classic classification algorithm, RF [18], which is an ensemble algorithm with multiple decision trees. In the training stage, each decision tree was grown from bootstrap samples [27] and random feature subsets [28]. In the bootstrap procedure, a training dataset containing *N* samples was repeatedly sampled for *B* times (B is the parameter representing the number of decision trees). For each decision tree, the randomly selected *N* samples with replacement constituted its training set, and a random feature subset was adopted to split the nodes of this decision tree. *B* decision trees were eventually grown. For a new sample, each decision tree provided a predicted class, and the predicted class of the RF was determined by majority voting. RF has been applied to tackle many biological problems [29,30,31,32,33,34,35,36].

The classifiers named “RandomForest” in Weka [37] with default parameters were used to build classification models.

### 2.5. Performance Evaluation

As mentioned in Section 2.1, AFPs were termed as positive samples, whereas non-AFPs were deemed as negative samples. By dealing with such a binary classification problem, some essential domains and one efficient classifier can be obtained. For binary classification, the following entries are always counted. They are true positive (TP), false positive (FP), false negative (FN), and true negative (TN), where TP/TN stands for the number of correctly predicted positive/negative samples, FN/FP denotes the number of incorrectly predicted positive/negative samples. Based on these entries, four measurements: sensitivity (SN), specificity (SP), accuracy (ACC), and Matthews correlation coefficient (MCC) can be computed. Their formulations are as follows:

(3) $$\mathrm{SN}=\frac{\mathrm{TP}}{\mathrm{TP}+\mathrm{FN}}$$

(4) $$\mathrm{SP}=\frac{\mathrm{TN}}{\mathrm{TN}+\mathrm{FP}}$$

(5) $$\mathrm{ACC}=\frac{\mathrm{TP}+\mathrm{TN}}{\mathrm{TP}+\mathrm{TN}+\mathrm{FP}+\mathrm{FN}}$$

(6) $$\mathrm{MCC}=\frac{\mathrm{TP}\;\times \mathrm{TN}-\mathrm{FP}\;\times \mathrm{FN}}{\sqrt{\left(\mathrm{TP}+\mathrm{FP})(\mathrm{TP}+\mathrm{FN})(\mathrm{TN}+\mathrm{FP})(\mathrm{TN}+\mathrm{FN}\right)}}$$

SN is the accuracy for positive samples, whereas SP is the accuracy for negative samples. In addition, ACC and MCC can fully evaluate the performance of classifiers. Considering the fact that MCC is a balanced measurement that is more accurate than ACC when the dataset is imbalanced, it is picked up as the key measurement in this study. The higher the above measurements are, the higher the performance of the classifier is.

## 3. Results

**Figure 1:**
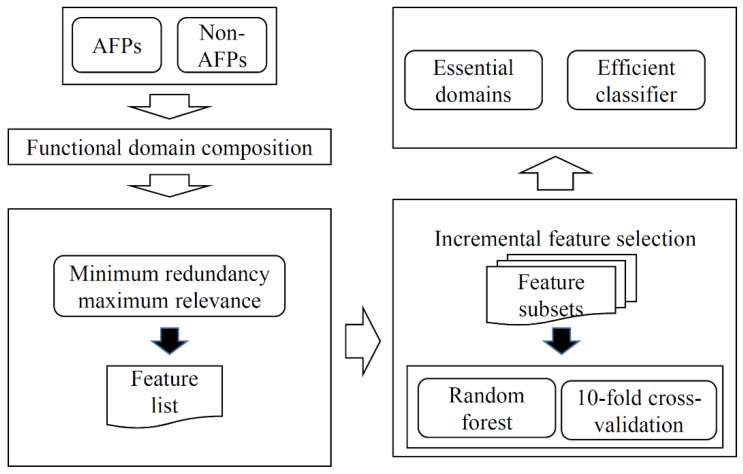
Entire procedures to construct the classifier to identify antifreeze proteins (AFPs). Each protein is represented by its functional domain composition. Then, the minimum redundancy maximum relevance method is used to analyze domain features, resulting in a feature list. Such a list is fed into the incremental feature selection, incorporating random forest (RF) as a classification algorithm, to construct an optimum RF classifier and extract essential domains.

In this study, we used functional domains to represent each protein and proposed an RF classifier to identify AFPs. Furthermore, some essential domains were extracted. The entire procedures are illustrated in Figure 1.

### 3.1. Results of mRMR Method

13729 domains were used to represent AFPs and non-AFPs. We first used the mRMR method to evaluate the importance of all features, obtaining an mRMR feature list. Such a list is provided in Supplemental Material S3.

### 3.2. Results of IFS Method

**Figure 2:**
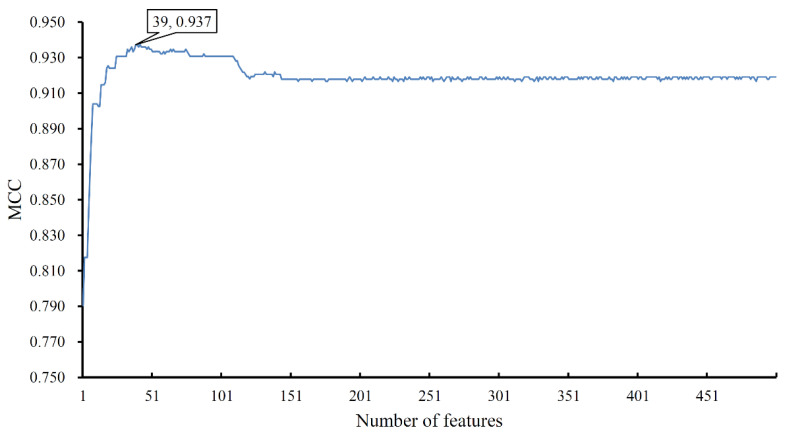
IFS curve to show the performance of random forest with a different number of features. The highest MCC is 0.937 when the top 39 features are used.

The mRMR feature list was fed into the IFS method. Because lots of domain features were involved, the IFS method would be time-consuming if all possible feature subsets were considered. To save time, we only constructed the top 500 feature subsets and built an RF classifier on each subset. All classifiers were evaluated by 10-fold cross-validation. Predicted results were counted as four measurements computed by Equations (3)–(6), which are available in Supplementary Material S4. For easy observation, an IFS curve was plotted in Figure 2, in which the number of features was set as *X*-axis and MCC was set as *Y*-axis. It can be observed that such a curve first followed an increasing trend and after it achieved the highest point, it followed a slightly decreasing trend. The highest MCC was 0.937, which was obtained by using the top 39 features. Accordingly, these 39 features were termed optimum features and the RF classifier with them was called the optimum RF classifier. The other three measurements (SN, SP, ACC) of such a classifier were 0.890, 1.000, and 0.995, respectively. These results indicated the good performance of such an RF classifier.

### 3.3. Comparisons with Previous Methods

The performance of the proposed RF classifier was compared with those of other state-of-the-art methods to reveal its advantages. Given that AFP-Ensemble [11] does not calculate MCC, only the measurements reported by both methods were included. As shown in Table 1, our classifier yielded the best performance across two measurements, namely, specificity and accuracy. In detail, the specificity and accuracy were at least 6% and 5.7%, respectively, higher than those obtained by other methods. In addition, our classifier used only 39 features, which is fewer than the 156 features used in AFP-Ensemble [11]. These results indicated that our proposed classifier showed satisfactory potential for AFP prediction.

With the above arguments, the proposed classifier was superior to previous methods. The most previous methods directly extracted features from protein sequences, which contained limited function information of proteins. The functional domain is much more powerful to identify different properties of proteins because it directly contains the function information of proteins. Although AFP-Ensemble [11] also employed functional domain to represent proteins, it only adopted fifteen domains which were annotated to no less than ten proteins. In fact, those domains that were annotated to few proteins may still be important to identify AFPs. Among 39 optimum features (domains), 15 (~38.46%) domains were annotated to less than 10 AFPs or non-AFPs. We employed all domains and adopted an advanced feature selection method to extract essential domains, which were further used to construct the proposed RF classifier. It is an important reason why our classifier was much better than AFP-Ensemble and other methods.

## 4. Discussion

**Figure 3:**
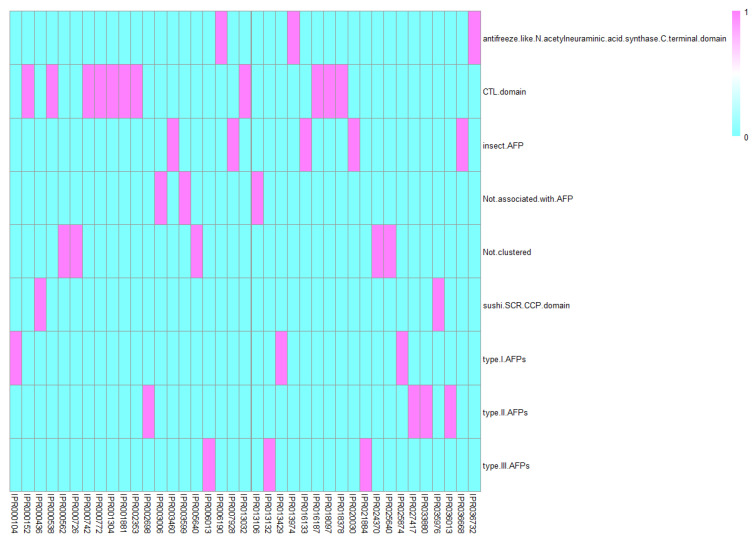
A heat map to show the classification of domains in each subgroup.

In the proposed RF classifier, each protein was represented by 39 features. These features were derived from 39 functional domains. According to the construction of such a classifier, these domains were highly related to AFPs. They were classified into seven subgroups for analysis: (1) CTL domain, (2) sushi/SCR/CCP domain, (3) antifreeze-like/N-acetylneuraminic acid synthase C-terminal domain, (4) insect AFP, (5) type I AFPs, (6) type II AFPs, and (7) type III AFPs. The detailed structure information link from the Interpro database and the cluster of each identified domain are provided in Supplementary Material S5. A heat map demonstrated the classification of domains in each subgroup is presented in Figure 3. Detailed analyses of each group of features can be seen below.

### 4.1. CTL Domains

CTL domain was matched by eleven InterPro entries and therefore is an important feature for AFP detection. CTLs are generally made up of 110–130 residues. The four typical cysteins are conserved at the structural level and involve a functional two-disulfide bond. Such conserved domains are shared in multiple protein subtypes. As a general cluster of AFPs, type II AFPs have been confirmed to be homologous to such calcium-dependent CTLs in a widely cultivated fish named Osmerus mordax [40]. Therefore, this structural feature may be crucial for the identification of novel, functional AFPs.

AFPs were assumed to have derived from lectin-associated residues in vivo [41]. The conserved lectin-associated genes exhibit function loss in various species as not-favored genes. These results show that the gene transfer between eukaryotes occurs naturally, suggesting that the ancestor gene of AFPs may have originated from the gene of the CTL domain [41].

Some typical peptide sequences and spatial structures do participate in the inhibition of cellular freezing and thus may partially reveal potential AFP mechanisms [40]. For instance, calcium-dependent ice recrystallization inhibition (IRI) is a functional biological regulatory mechanism for antifreeze properties in plants and also involves CTL domains [40]. Some plant CTLs change the Ca^2+^ concentration to affect the IRI activity without inducing sequence homology to AFPs. Hence, additional non-AFPs may exist with the ability to display calcium-dependent ice recrystallization inhibition.

Among these domains, three of them are just c-type lectin-like domains (IPR001304, IPR016187, IPR018378), which are CTL domains. Apart from that, some additional domains like link domain (IPR000538), EGF associated domains (IPR001881, IPR000742, IPR000152, IPR013032, IPR018097), and a lectin associated domain have also been clustered into CTL domains. Considering the anti-freeze capacity of CTL domains and EGF domains have been validated in Section 1, it is reasonable for us to identify such terms as anti-freeze protein-associated domains.

### 4.2. Sushi/SCR/CCP Domains

Sushi/SCR/CCP domain was found by two InterPro entries in numerous complement and adhesion proteins. This domain has typical structural properties that reflect its effective biological functions. For each CCP domain, approximately 60 residues have formed a similar domain with CTL [42,43]. Considering the correlations between structural and functional similarities, the sushi/SCR/CCP domain may also display calcium-dependent IRI, thereby corresponding with our prediction result. As for the detailed terms, two IPR terms describing Sushi/SCR/CCP domain (IPR000436) and Sushi/SCR/CCP superfamily (IPR035976) have been identified, which are quite reasonable.

### 4.3. Antifreeze-Like/N-Acetylneuraminic Acid Synthase

Antifreeze-like/N-acetylneuraminic acid synthase C-terminal domain, also called antifreeze-like protein (AFLP), is an important feature to differentiate AFPs from other proteins. AFLPs are similar to AFPs. Early in 2006, the structure of AFLP was identified and validated by one of the most advanced techniques, nuclear magnetic resonance spectroscopy. Composed of 75 amino acid residuals, the AFL domain is the core functional residuals for AFLPs [44]. Similar structural and sequential properties were found between AFLPs and type III AFPs; however, their biological stability and potential regulatory mechanisms differ under alternative temperatures [5]. Therefore, these two groups were separately classified despite their structural similarity [45]. AFLPs are also widely distributed under natural conditions. Considering their potential functions and distinctive antifreeze performance, AFLPs must be classified into a particular AFP category. The three IPR terms: SAF domain (IPR013974), antifreeze-like/N-acetylneuraminic acid synthase C-terminal (IPR006190), and Antifreeze-like/N-acetylneuraminic acid synthase C-terminal domain superfamily (IPR036732), are included in this subgroup. The last two terms just describe the antifreeze-like/N-acetylneuraminic acid synthase C-terminal domain associated structures. As for the SAF domain, it is also a member of the antifreeze-like/N-acetylneuraminic acid synthase C-terminal domain superfamily.

### 4.4. Insect AFPs

Insect AFP was matched by five InterPro entries and identified in insects. The core regulatory mechanism for insect AFP is the regulation of freezing points on aqueous solutions [46]. For instance, the AFPs in D. canadensis contain effective ice-binding sites [9]. The in vivo insect AFPs accumulate at the air-water interface [9], thus indicating their antifreeze capacity. Therefore, this typical subtype of AFPs with species-specific distribution and functions can be categorized as a particular AFP subgroup. Three terms have a direct description as insect-associated AFPs (IPR016133, IPR036668, IPR003460). As for another two terms, both of them are associated with specific types of insects (IPR007928 and IPR020030).

### 4.5. AFP, Type I

Type I, II, and III AFPs are the most common subtypes. Type I AFPs are a group of gas hydrate crystal inhibitors, and their antifreeze ability relies on their binding capacity against the ice plane. Type I AFPs contribute to the regulation of intracellular antifreeze biological processes as confirmed in multiple artificial and natural environments [47,48], indicating that the features of such a group may help identify novel AFPs. Furthermore, the biological functions of type I AFPs are correlated with and contribute to the local melting of ice upon adsorption to surfaces [47,48], thus reflecting a special regulatory mechanism for this subtype. Therefore, type I AFPs have specific antifreeze regulatory effects and mechanisms compared with other AFPs, thus validating the efficacy and accuracy of our feature subgrouping. For type I AFP, only zinc ribbon-associated anti-freeze proteins (IPR013429 and IIPR025874) and a general description of antifreeze protein, type I (IPR000104) have been identified.

### 4.6. AFP, Type II

Compared with type I AFPs, type II AFPs have different natural distribution patterns and are found in species such as sea ravens, smelt, and herring. With regard to structure, type II AFPs can also be regarded as the evolved CTLs with calcium ion-dependent functions. In a natural environment, the antifreeze capacity of herring and smelt can be attributed to type II AFPs [49], indicating the biological basis of our feature classification. On the basis of the evolutionary routines of CTL domains, the type II AFP gene is suggested to be a typical and complicated evolved antifreeze subgroup from the classic CTLs [49]. Under differentiated selection pressures, type II AFPs share some common properties with the original CTLs and also contain some specific and unique structures, thus forming a new subgroup of antifreeze proteins. For instance, some type II AFPs such as those in sea ravens can remove the restriction of calcium ions and constitute a new non-calcium-reliant mechanism for the antifreeze processes [49]. Due to the complicated antifreeze structures, some of the identified terms of type II AFPs have been fully described, but the biological functions have not been revealed, including SPFH domain, Ydjl-like (IPR033880), Band7/SPFH domain superfamily (IPR036013), 5-formyltetrahydrofolate cyclo-ligase (IPR002698) and P-loop containing nucleoside triphosphate hydrolase (IPR027417).

### 4.7. AFP, Type III

Different from type II AFPs that have evolved from CTLs, type III AFPs are derived from a sialic acid synthase gene according to an evolutionary genomic study on Antarctic eelpout [5]. A group of complicated biological structure signatures, including multiple α-helix, helices, and β-strands, have been identified in type III AFPs [5]. The first typical type III AFP was purified from a viviparous European eel named Zoarces viviparus [50]. Two isoforms of type III AFPs, namely, QAE1 (ZvAFP13) and SP (ZvAFP6) were also identified in such species, thus reflecting the diversity of this subtype. For their functional mechanisms, type III AFPs can promote the meiotic spindle morphology and the fertilization and blastocyst rates of sperm cells and embryos under freezing conditions [51]. According to a series of studies on the protective role of type III AFPs in reproductive cells [51,52,53], this subtype can protect the cells, especially reproductive cells, from freezing to death and provide maximum maintenance on their reproductive capacity. Therefore, type III AFPs have also been applied to modify and evaluate sperm cryopreservation. A general description of antifreeze protein type III (IPR006013) and two summarized type III AFPs according to Pfam classification (Ice-binding protein as IPR021884, N-acetylneuraminic acid synthase, N-terminal as IPR013132) have been identified and predicted to be associated with AFPs.

Previous works have confirmed these predicted high-ranking sequence features (motifs) of AFPs, thus conforming to our prediction. Therefore, the present study has developed a novel computational approach for the detection of potential AFPs and helps us partially reveal the common structural characteristics of anti-freeze associated proteins.

## 5. Conclusions

This study used functional domains to represent each AFP or non-AFP. Then, some machine learning methods were applied to identify AFP-associated protein domains. According to recent publications, several identified domains with reliable annotation are associated with seven clusters of AFPs. These results can help us take the initial step to understand the biological mechanisms of AFPs at the structural level. Moreover, the classifier using identified domains provided better performance than some previous methods, which can be a useful tool for identifying latent AFPs.

## Figures and Tables

**Table 1:** Performance comparison of different methods for classifying AFPs.

Method	MCC	Sensitivity ^#^	Specificity ^#^	Accuracy ^#^
Our classifier	0.937	0.890	**1.000**	**0.995**
AFP-Pred [6] *	-	0.847	0.840	0.843
AFP-PSSM [38] *	-	0.759	0.933	0.930
AFP-PseAAC [39] *	-	0.862	0.847	0.848
AFP-Ensemble [11] *	-	**0.892**	0.940	0.938

*: The results of the other four methods are from [11]. ‘-’ indicates that this measurement is not reported in their methods. ‘#’: Bold number in one column indicates the highest value in the corresponding column.

## Supplementary Materials

The following are available online at https://www.mdpi.com/article/10.3390/life11060520/s1, Supplementary Material S1: AFP and non-AFP sequences, Supplementary Material S2: Protein domain matrix, Supplementary Material S3: mRMR results, Supplementary Material S4: Prediction performances, Supplementary Material S5: Detailed structure information link from Interpro database and the cluster of each identified domain.
